# Phrenic nerve injury with pulsed field ablation: A lesson to be learned?

**DOI:** 10.1016/j.hrcr.2024.04.013

**Published:** 2024-05-03

**Authors:** Joonhyuk Kim, David Slotwiner

**Affiliations:** New York Presbyterian/Queens, Flushing, New York

Catheter ablation therapy is the most effective method of maintaining sinus rhythm in patients with atrial fibrillation. The first ablation modality was direct current ablation and was used for ablation of the atrioventricular node.^1^ Then came thermal ablation (radiofrequency and cryothermal [CT]), and with it, there were improvements in efficacy and safety. This allowed for more complex techniques such as pulmonary vein isolation (PVI). However, neither is completely effective or completely safe, and some difficult *real-world* lessons were learned as experience increased and adoption became widespread. An example is the first reports of atrioesophageal fistula 12 years after PVI was described.^2^ Now, the era of nonthermal ablation has started with the introduction of pulsed field ablation (PFA). It holds the promise of further improvement in safety, but only once a new technology becomes widely used in clinical practice can its true *real-world* safety and efficacy be assessed.

In this issue of *Heart Rhythm Case Reports*, Franceschi and colleagues^3^ report a case of a 75-year-old man who underwent ablation of atrial fibrillation (PVI and ablation of the posterior and superior left atrial walls) with PFA. During ablation at the right superior pulmonary vein, phrenic nerve injury (PNI) was noted. The authors elegantly describe the progressive reduction in compound motor action potential amplitude with each PFA application near the right superior pulmonary vein. Eventually, there was complete loss of diaphragmatic contraction. Then, incomplete recovery of phrenic nerve function both by compound motor action potential and tactile contraction was noted, prior to completion of the ablation. The patient was asymptomatic and with a normal chest radiograph the next day.

The mechanism of cell death from PFA depends on high-amplitude electrical fields, delivered in pulses, to cause increased permeability and injury to the cell membrane. The term for this is electroporation and its permanency is dependent on the strength of the electrical field. There may be irreversible cell death from electroporation or reversible cellular injury.^4^ The effects are tissue specific and since there is little or no thermal injury, the adjacent tissue or extracellular matrix is not affected.^5^ Therein lies the promise of increased safety.

With all catheter ablation therapy, there is risk of mechanical vascular or cardiac injury, systemic embolization of thrombus, or infection. However, there are risks that are specific to thermal ablation. Fortunately, the risks are rare, but pericarditis, pulmonary vein stenosis, PNI, and atrioesophageal fistula are all associated with both radiofrequency and CT ablation.^6^ On the contrary, since PFA is tissue specific, there is anticipation that those risks could be reduced or eliminated. With specific regard to the purported adverse effect in this case, the ADVENT trial provides reassurance. This study compared PFA to thermal ablation in ablation of atrial fibrillation and showed noninferiority in efficacy in arrhythmia recurrence with no difference in serious adverse effects. No PNI was seen in the PFA arm, whereas 2 subjects in the thermal ablation arm (both were CT ablation with a balloon catheter) had PNI.^7^ In an earlier study, injury of the right phrenic nerve was not seen despite PFA in the superior vena cava in an attempt to purposely injure that nerve.^8^ The result of these reassuring data is that phrenic nerve conduction is not monitored during PFA, as is the case with CT ablation.^9^

On the other hand, the MANIFEST PF Registry suggested that PNI was possible. That study found that 0.4% of patients undergoing PFA had transient PNI and 1 patient out of the 1568 subjects had persistent PNI.^10^

PFA is a disruptive technology that is poised to change how electrophysiologists manage arrhythmias. The mechanism of how PFA affects the phrenic nerve is not yet clear, but the case by Franceschi and colleagues serves as a reminder that as PFA is becoming widely available, clinicians will only now be able to evaluate its *real-world* safety and efficacy and that the promises of a novel therapy should be embraced with vigilance.

## Disclosures

There are no conflicts of interest.
